# Comparative Evaluation of Aluminum Sulfate and Ferric Sulfate-Induced Coagulations as Pretreatment of Microfiltration for Treatment of Surface Water

**DOI:** 10.3390/ijerph120606700

**Published:** 2015-06-12

**Authors:** Yali Song, Bingzhi Dong, Naiyun Gao, Yang Deng

**Affiliations:** 1 School of Civil Engineering and Architecture, Zhejiang University of Science and Technology, Hangzhou 310023, China; E-Mail: yali_song@sina.com; 2 College of Environmental Science and Engineering, Tongji University, Shanghai 200092, China; E-Mail: gaonaiyun@sina.com; 3 Department of Earth and Environmental Studies, Montclair State University, Montclair, NJ 07043, USA; E-Mail: dengy@mail.montclair.edu

**Keywords:** coagulation, microfiltration, membrane flux, organic matter, membrane fouling

## Abstract

Two coagulants, aluminum sulfate and ferric chloride, were tested to reduce natural organic matter (NOM) as a pretreatment prior to polyvinylidene fluoride (PVDF) microfiltration (MF) membranes for potable water treatment. The results showed that the two coagulants exhibited different treatment performance in NOM removal. Molecular weight (MW) distributions of NOM in the tested surface raw water were concentrated at 3–5 kDa and approximately 0.2 kDa. Regardless of the coagulant species and dosages, the removal of 0.2 kDa NOM molecules was limited. In contrast, NOM at 3–5 kDa were readily removed with increasing coagulant dosages. In particular, aluminum sulfate favorably removed NOM near 5 kDa, whereas ferric chloride tended to reduce 3 kDa organic substances. Although aluminum sulfate and ferric chloride could improve the flux of the ensuing MF treatment, the optimal coagulant dosages to achieve effective pretreatment were different: 2–30 mg/L for aluminum sulfate and >15 mg/L for ferric chloride. The scanning electron microscope (SEM) image of the membrane-filtered coagulated raw water showed that coagulation efficiency dramatically affected membrane flux and that good coagulation properties can reduce membrane fouling.

## 1. Introduction

During the past few decades, low-pressure-driven membrane processes such as microfiltration (MF) and ultrafiltration (UF) have emerged as new drinking water treatment technologies owing to their high treatment efficiency, simple operation, and small physical footprint. However, fouling is the major barrier to application of these membrane processes in practice. Prior studies well demonstrate that membrane fouling is principally caused by colloids and dissolved organic matter (e.g., humic acid) in potable water treatment [1].

To minimize membrane fouling, a variety of pretreatment methods have been studied, such as coagulation, adsorption, and chemical oxidation [2,3,4]. Among the pretreatment options, coagulation is preferred owing to the following reasons: (1) simple operation, (2) low cost, and (3) capability to remove particulates and NOM from water, all of which are well recognized as major causes of membrane fouling [5,6]. Therefore, coagulation has become one of the most frequently used pretreatments prior to low pressure-driven membrane filtration [7,8,9,10].

Aluminum (Al) and iron (Fe) salts are two widely used coagulants. Large flocs are formed in water subsequent to hydrolysis of the metal-based coagulants and then settled in a sedimentation tank. The floc properties (e.g., size, density, and strength) determined by coagulant species, chemical dosage, and solution chemistry significantly influence coagulation efficiency. Further, the coagulation performance can greatly affect the membrane process [11,12,13,14]. For example, small floc sizes lead to high cake resistance on membranes [15,16]. Prior efforts were made to determine an optimal coagulant dosage to minimize membrane fouling [17,18,19]. Lee *et al.* found an optimal polyaluminum chloride (PACl) dosage with respect to fouling minimization, and the dosage depended heavily on the physical and chemical characteristics of the wastewater [20]. Tran *et al.* reported that dissolved organic carbon (DOC) in water could be significantly reduced with a specified dosage of Al to reduce membrane fouling [21]. 

The objective of this study was to evaluate two common coagulants, aluminum sulfate and ferric chloride, as pretreatments prior to MF treatment. Particularly, the permeate flux decline and the role of NOM were studied. 

## 2. Experimental Methods

### 2.1. Reagents and Materials

Raw water was collected from the Huangpu River (Shanghai, China). This river is a major drinking water source for the city of Shanghai. All chemicals used were at least analytical grade except as noted. Aluminum sulfate (Al_2_(SO_4_)_3_·18H_2_O) and ferric chloride (FeCl_3_·6H_2_O) were used as coagulants. Hollow fibrous MF membranes were provided from Toray, Japan. Basic physical and chemical characteristics of the membranes are summarized in Table 1.

**Table 1:** Basic physical and chemical parameters of the microfiltration (MF) membranes used.

Material	Polyvinylidene fluoride (PVDF)
Type	Hollow fiber
Pore size, μm	0.1
Membrane surface area, cm^2^	75
Type of filtration	Dead-end
Type of pressure	Outside-inside

### 2.2. Coagulation Pretreatment

Coagulation pretreatment tests were conducted in 1-L beakers on a classical jar testing apparatus. Appropriate amounts (2–30 mg/L) of Al_2_(SO_4_)_3_·18H_2_O or FeCl_3_·6H_2_O were added to 1000 mL raw water in the beakers to initiate the coagulation process. Rapid mixing at 100 rpm proceeded for 1 min, followed by 30 min of slow mixing at 30 rpm. Thereafter, sedimentation for 30 min allowed large flocs to settle down. Finally, the supernatant was collected and used for the following membrane filtration.

### 2.3. Experimental Apparatus

**Figure 1:**
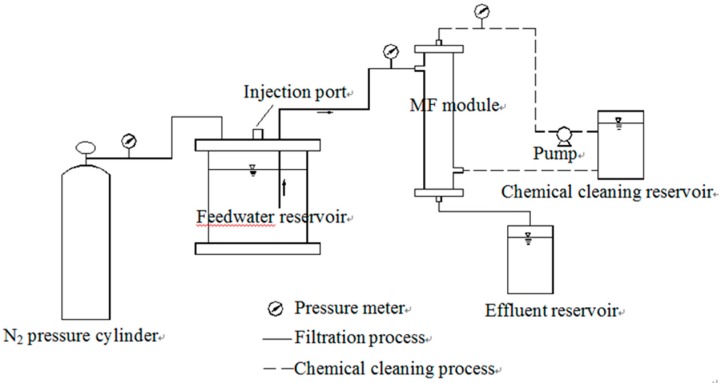
Schematic of the experimental setup.

The MF treatment setup is shown in Figure 1. The apparatus was composed of a N_2_ pressure cylinder, a feedwater tank and a membrane module. Nitrogen gas from the N_2_ pressure cylinder forced the coagulated water stored in the feedwater tank through the membrane in the MF module. Rapid mixing by a magnetic stirrer ensured that the water sample achieved a complete mixing state in the feedwater reservoir. After every 800 mL water sample was filtered, chemical cleaning was applied to mitigate membrane fouling. Chemical cleaning included two successive steps: 2 h of 1% oxalic acid cleaning and 2 h of 5000 mg/L sodium hypochlorite cleaning. 

### 2.4. Analysis

Measure of molecular weight fractionation, similar to the procedure described by Hu *et al.* [22], was conducted using an HPLC system consisting of a LC-10AD pump (Shimadzu), an SPD-20A UV detector (Shimadzu), a SCL-10A system controller (Shimadzu, Tokyo, Japan) and a G2500PWXL column (TSK). The mobile phase was 0.05 mol/L sodium sulfate with a flow rate of 0.5 mL/min. Polystyrene sulphonates (PSS) standards of MW 14, 7.5, 4, 1.5, 0.7, 0.5, and 0.2 kDa were used to calibrate the system. The MW distribution results were analyzed based on the response (volt) data over the elapsed time. 

After the samples were filtered through 0.45 μm membranes, dissolved organic carbon (DOC) was analyzed with a TOC analyzer (Shimadzu TOC-Vcpn). UV absorbance at 254 was measured using a UV spectrophotometer (HACH DR5000). Scanning electron microscope (SEM) imaging was conducted in ESEM (PHILIPS XL30, Amsterdam, The Netherlands).

## 3. Results and Discussion

### 3.1. Molecular Weight Distribution of Dissolved Organic Matter (DOM)

**Figure 2:**
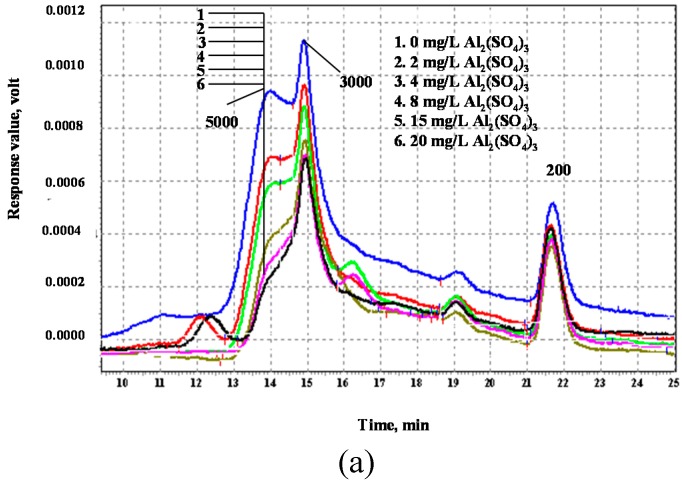

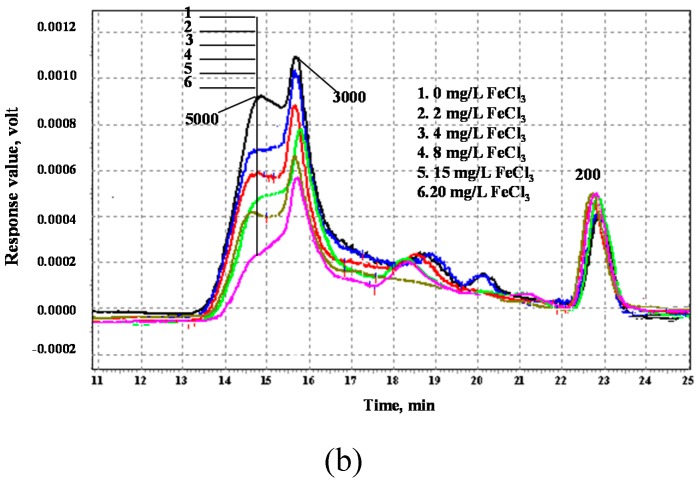
(**a**) Effect of aluminum sulfate on MW of organic matter; (**b**) Effect of ferric chloride on MW of organic matter.

The molecular weight distribution of the DOM before and after aluminum sulfate and ferric chloride treatment is shown in Figure 2a,b. DOM in the raw water was concentrated within 3–5 kDa and near 0.2 kDa. Generally, the DOM was reduced to different degrees with increasing dosages of aluminate sulfate or ferric chloride. A low dosage of aluminum sulfate (e.g., 2 or 4 mg/L Al) slightly removed DOM within 3–5 kDa. However, a further increase of the chemical dosage to 8–20 mg/L significantly removed DOM in this range. In contrast, the removal of DOM near 0.2 kDa was minor, regardless of the aluminum sulfate dosage. This finding is ascribed to the following reasons. The dominant coagulation mechanism for DOM removal is associated with electrical neutralization as well as adsorption of DOM on the coagulants’ hydrolytic products. High-MW DOM molecules (3–5 kDa DOM in this study) are mostly hydrophobic compounds that coagulation preferentially removes. In contrast, the major fraction of low-MW compounds (0.2 kDa in this study) characterized by hydrophilic properties is only slightly removed by these coagulants. Generally, ferric chloride exhibited a similar removal pattern. 

As seen in Figure 2, aluminum sulfate considerably removed the DOM molecules near 5 kDa molecular weight. The significant removal of 3–5 kDa DOM occurred at a chemical dose of 8 mg/L. However, the increase in coagulant dosage only slightly improved the removal at >8 mg/L aluminum sulfate. In contrast, the DOM removal continued to increase with increasing ferric chloride dosage. Particularly, ferric chloride removed more organic matter near 3 kDa MW than aluminum sulfate. 

### 3.2. DOC and UV_254_

**Figure 3:**
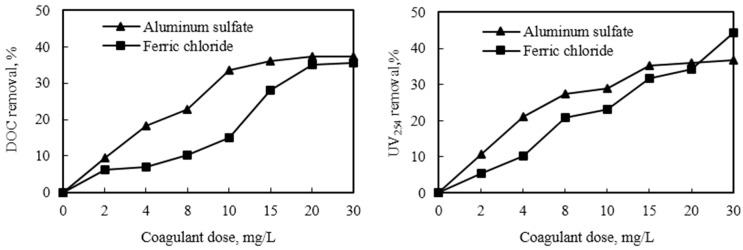
Effect of different coagulants on dissolved organic carbon (DOC) and UV_254_ removals.

The reduction of DOC and UV_254_ by aluminum sulfate and ferric chloride is shown in Figure 3. When the aluminum sulfate dosage was increased from 2 to 30 mg/L, the removal rates of DOC and UV_254_ gradually augmented from 9.5% to 37.4% and from 10% to 37%, respectively. Interestingly, the removal rates sharply increased at a 2–15 mg/L coagulant dose but slowed down at >15 mg/L. This is similar to the results found by Zhou *et al.* [23]. 

When the ferric chloride dosage was increased from 2 to 30 mg/L, the removal rates of DOC and UV_254_ were increased from 6.3% to 35.6% and from 5.5% to 44%, respectively. At any specific dosage, alum removed more DOC and UV_254_, except at 30 mg/L under which the UV_254_ removal by ferric chloride dramatically increased over the removal by alum. Zhou *et al.*, found that ferric chloride effectively removed double bonds or aromatic ring unsaturated compounds [23].

### 3.3. Membrane Flux

The membrane fouling decline rate was employed to evaluate the variation of membrane flux with different coagulants. The membrane fouling decline rate (Φ) can be calculated as follows:

(1) $$\text{Φ}\;=\;\frac{\left(J_{C}-J_{R}\right)}{\left(100-J_{R}\right)}\times 100$$

Φ—membrane fouling decline rate, %
*J_R_*—final membrane flux of raw water, %
*J_C_*—final membrane flux of coagulation treatment, %

**Figure 4:**
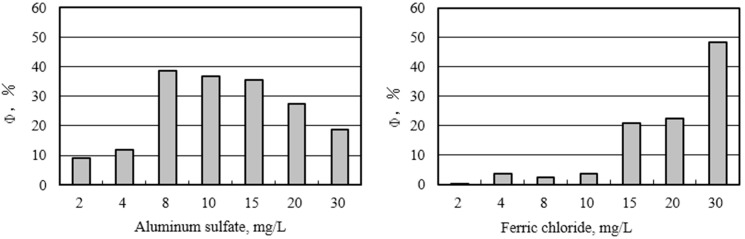
Effect of different coagulation pretreatments on membrane fouling decline.

The variation of membrane fouling decline rate using different coagulants is shown in Figure 4. Aluminum sulfate and ferric chloride exhibited different behaviors. For aluminum sulfate, Φ increased from 9.1% to 38.5% with increasing dosage from 2 to 8 mg/L and then gradually decreased to 18.5% when the dosage was further increased to 30 mg/L. In contrast, for ferric chloride, as the dosage was increased from 2 to 30 mg/L, Φ constantly increased from 0.2% to 22.2%. At 2–20 mg/L, aluminum sulfate caused a greater Φ than ferric chloride. However, at 30 mg/L, Φ in ferric chloride was far higher than that in aluminum sulfate. These findings suggest that the optimal alum dosage existed to achieve a high Φ because poor hydrolyzation at a high coagulant dosage led to formation of small flocs and poor settling efficiencies of flocs in feedwater, thereby increasing membrane resistance. However, higher dosages caused higher Φ when ferric chloride was applied. 

The different Φ patterns of membrane fouling decline of aluminum sulfate and ferric chloride are likely associated with the formation of flocs. Prior studies demonstrated that flocs would promote the reduction in membrane resistance and improve the membrane flux [24,25,26,27]. Dong also reported that membrane flux was closely related to the size of flocs. At the optimal coagulant dosage, the largest floc was formed with the best settling [28]. In this study, 8 mg/L aluminum sulfate produced the biggest floc and resulted in the greatest floc settling. However, when the aluminum sulfate dosage was too high (>8 mg/L), a large quantity of small flocs was produced with very poor settling properties. The small would accumulate on the membrane and form a compacted cake layer, thus increasing the filtration resistance. In contrast, the optimal dosage was not observed at 2–30 mg/L ferric chloride. Large and compacted flocs on the membrane did not reduce the membrane fouling decline rate.

### 3.4. Discussion 

**Figure 5:**
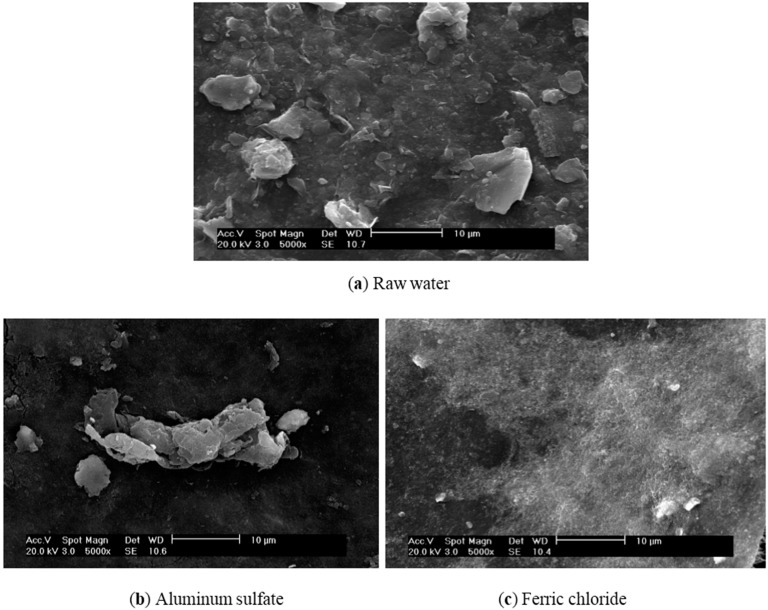
SEM images of membrane surface after different coagulations and filtration (×5000).

Prior intensive efforts demonstrate that NOM is a major contributor to membrane fouling [29,30,31]. Coagulation, if performed well, can dramatically reduce membrane fouling. The SEM images of membranes under different operational conditions are shown in Figure 5. Many deposits were observed on the membrane (Figure 5a) when the MF directly filtered the raw water. However, the membrane surface was much cleaner with a few large deposits after aluminum sulfate-induced coagulation was applied (Figure 5b). In contrast, more uniform deposits were observed on the membrane surface with ferric chloride coagulation pretreatment (Figure 5c) because smaller and more compacted flocs were formed by ferric chloride coagulation. These images again provide evidence of why aluminum sulfate prevents membrane fouling better than ferric chloride.

## 4. Conclusions

Coagulation is a viable pretreatment option prior to MF treatment for water from the Huangpu River. It preferentially removes high-MW dissolved organic matters that tend to form compacted cake layers on membrane surfaces. Either aluminum sulfate or ferric chloride appears to be a good coagulant, but they perform in different patterns of membrane fouling decline rate. The optimal dosage for membrane fouling flux was found to be 2–30 mg/L aluminum sulfate, whereas membrane fouling decline was improved gradually by the increase of ferric chloride from 2 mg/L to 30 mg/L. This also indicates that the results of coagulation have a great effect on membrane fouling material.

## References

[1] Takizawa S., Fujita K., Soo K.H. (1996). Membrane fouling decrease by microfiltration with ozone scrubbing. Desalination.

[2] Sakol D., Konieczny K. (2004). Application of coagulation and conventional filtration in raw water pretreatment before microfiltration membranes. Desalination.

[3] Park P.-K., Lee C.-H., Choib S.-J., Choo K.-H., Kimd S.-H., Yoone C.-H. (2002). Effect of the removal of DOMs on the performance of a coagulation-UF membrane system production. Desalination.

[4] Konieczny K., Klomfas G. (2002). Using activated carbon to improve natural water treatment by porous membranes. Desalination.

[5] Taniguchi M., Kilduff J.E., Belfort G. (2003). Modes of natural organic matter fouling during ultrafiltration. Environ. Sci. Technol..

[6] Huang H., Spinette R., O’Melia C.H. (2008). Direct-flow microfiltration of aquasols I. Impacts of particle stabilities and size. J. Memb. Sci..

[7] Kabsch-Korbutowicz M. (2005). Application of ultrafiltration integrated with coagulation for improved NOM removal. Desalination.

[8] Cho M.-H., Lee C.-H., Lee S. (2006). Effect of flocculation conditions on membrane permeability in coagulation–microfiltration. Desalination.

[9] Konieczny K., Sąkol D., Bodzek M. (2006). Efficiency of the hybrid coagulation–ultrafiltration water treatment process with the use of immersed hollow-fiber membranes. Desalination.

[10] Konieczny K., Bodzek M., Rajca M. (2006). A coagulation-MF system for water treatment using ceramic membranes. Desalination.

[11] Yu W.-Z., Liu H.-J., Liu T., Liu R.-P., Qu J.-H. (2013). Comparison of submerged coagulation and traditional coagulation on membrane fouling: Effect of avtive flocs. Desalination.

[12] Dong H., Gao B., Yue Q., Rong H., Sun S., Zhao S. (2014). Effect of Fe(III) species in polyferric chloride on floc properties and membrane fouling in coagulation-ultrafiltration process. Desalination.

[13] Xu W., Gao B., Mao R., Yue Q. (2011). Influence of floc size and structure on membrane fouling in coagulation–ultrafiltration hybrid process-the role of Al_13_ species. J. Hazard. Mater..

[14] Xu W., Gao B. (2012). Effect of shear conditions on floc properties and membrane fouling in coagulation/ultrafiltration hybrid process––The significance of Al_b_ species. J. Memb. Sci..

[15] Wang J., Pan S., Luo D. (2013). Characterization of cake layer structure on the microfiltration membrane permeability by iron pre-coagulation. J. Environ. Sci..

[16] Zhao B., Wang D., Li T., Chow C.W.K., Huang C. (2010). Influence of floc structure on coagulation-microfiltration performance: Effect of Al speciation characteristics of PACls. Sep. Purif. Technol..

[17] Ma B., Yu W., Liu H., Qu J. (2014). Effect of low dosage of coagulant on the ultrafiltration membrane performance in feedwater treatment. Water Res..

[18] Shon H.K., Vigneswaran S., Ngo H.H., Aim R.B. (2005). Is semi-flocculation effective as pretreatment to ultrafiltration in wastewater treatment?. Water Res..

[19] Mao R., Wang Y., Zhao Y., Gao B., Dong M. (2013). Impact of various coagulation technologies on membrane fouling in coagulation/ultrafiltration process. Chem. Eng. J..

[20] Lee B.B., Choo K.-H., Chang D., Choi S.-H. (2009). Optimizing the coagulant dose to control membrane fouling in combined coagulation/ultrafiltration systems for textile wastewater reclamation. Chem. Eng. J..

[21] Tran T., Gray S., Naughton R., Bolto B. (2006). Polysilicato-iron for improved NOM removal and membrane performance. J. Memb. Sci..

[22] Hu M., Lin J., Xu G., Dong B. (2013). Effect of relative molecular mass distribution and hydrophilicity/hydrophobicity of NOM on membrane fouling in MF-combined process. Environ. Sci..

[23] Zhou L., Zhang Y., Sun L., Li G. (2008). Characteristic of natural organic matter removal by ferric and aluminium coagulation. Environ. Sci..

[24] Najm I.N., Snoeyink V.L., Suidan M.T., Lee C.H., Richard Y. (1990). Effect of particle size and background natural organics on the adsorption efficiency of PAC. Am. Water Works Assoc..

[25] Rong H., Gao B., Li J., Zhang B., Sun S., Wang Y., Yue Q., Li Q. (2013). Floc characterization and membrane fouling of polyferric-polymer dual/composite coagulants in coagulation/ultrafiltration hybrid process. J. Colloid Interface.

[26] Zhao S., Gao B., Sun S., Yue Q., Dong H., Song W. (2015). Coagulation efficiency, floc properties and membrane fouling of polyaluminum chloride in coagulation–ultrafiltration system: The role of magnesium. Colloids Surf. A: Physicochem. Eng. Asp..

[27] Feng L., Wang W., Feng R., Zhao S., Dong H., Sun S., Gao B., Yue Q. (2015). Coagulation performance and membrane fouling of different aluminum species during coagulation/ultrafiltration combined process. Chem. Eng. J..

[28] Dong B.Z., Chen N.Y., Deng H.P., Fan J.C. (2005). Effect of coagulation on preventing membrane from fouling. Environ. Sci..

[29] Malgorzata K.K. (2006). Impact of pre-coagulation on ultrafiltration process performance. Desalination.

[30] Maria D.K., Kamanyi J., Heijman B.G.J., Gary A. (2008). Colloidal organic matter fouling of UF membranes: Role of NOM composition & size. Desalination.

[31] Huang H., Lee N., Young T., Gary A., Lozier J.C., Jacangelo J.G. (2007). Natural organic matter fouling of low-pressure, hollow-fiber membranes: Effects of NOM source and hydrodynamic conditions. Water Res..

